# Clinical Manifestations of Herpes Zoster, Its Comorbidities, and Its Complications in North of Iran from 2007 to 2013

**DOI:** 10.1155/2015/896098

**Published:** 2015-03-29

**Authors:** Farhang Babamahmoodi, Ahmad Alikhani, Fatemeh Ahangarkani, Leila Delavarian, Hamidreza Barani, Abdolreza Babamahmoodi

**Affiliations:** ^1^Antimicrobial Resistance Research Center, Department of Infectious Diseases, Mazandaran University of Medical Sciences, Sari, Iran; ^2^Health Management Research Center, Baqiyatallah University of Medical Sciences, Tehran, Iran

## Abstract

*Background*. Herpes zoster infection is a painful worldwide disease. Inappropriate and delayed treatment causes prolongation of the disease with debilitating symptoms and postherpetic neuralgia.* Method*. A cross-sectional study evaluated shingles cases admitted in a teaching hospital with one-year followup in north of Iran from 2007 to 2013.* Results*. From 132 patients, 60.4% were male. Head and neck involvement occurred in 78 people (59.1%), thoracoabdominal region in 37 cases (28%), and extremities in 16 cases (12.1%), and one case (0.8%) got multisites involvement. 54 cases (40.9%) had predisposing factors including diabetes mellitus in 26 cases (19.7%), malignancy in 15 (11.4%), immunosuppressive medication in 7 (5.03%), HIV infection in 3 (2.3%), radiotherapy in 2 (1.5%), and tuberculosis in one patient (0.8%). The most common symptoms were pain (95.5%), weakness (56%), fever (31.1%), headache (30.3%), ocular complaints (27.3%), itching (24.2%), and dizziness (5.3%). 21 cases (15.9%) had bacterial superinfection on blistering areas and overall 18 cases (13.6%) had opium addiction. 4 cases (3.03%) died during admission because of comorbidities. Postherpetic neuralgia was reported in 56 patients (42.5%) after three months and seven cases (5%) in one-year followup.* Conclusion.* Shortening interval between skin lesion manifestation and starting medication can accelerate lesion improvement and decrease disease course, extension, and complication.

## 1. Introduction

Chickenpox and herpes zoster are caused by varicella zoster virus (VZV), a DNA virus belonging to the herpes virus group. Primary infection with VZV causes chickenpox. after resolving of chickenpox, the VZV remains latent in the nerve cell bodies and less frequently the nonneuronal satellite cells of the dorsal root, cranial nerve, or autonomic ganglia. Herpes zoster, also known as shingles, results from reactivation of the latent infection [1].

There is considerable interest in the disease due to epidemiological variations between geographic location, especially between temperate and tropical regions of the world [2].

VZV remains latent in human nerve tissue and reactivates in approximately 15% to 30% of infected persons during their lifetime, resulting in herpes zoster (shingles). Shingles usually presents as a vesicular rash with pain and itching in a dermatomal distribution [3].

Shingles incidence increases with age, especially after the age of 50 and is more common among immunocompromised persons and among children with a history of intrauterine varicella or varicella occurring within the first year of life; the latter have increased risk of developing shingles at an earlier age [4].

Diagnosis of herpes zoster depends on clinical pictures and laboratory confirmation is not usually indicated. Serologic testing is not helpful. Serology of exposed contacts is not routinely recommended, although it may be requested in certain circumstances (e.g., for pregnant women and other high-risk contacts, and in healthcare settings) [5].

The most striking feature of shingles is the increase in incidence found with increasing age. Decreasing cell mediate dimmunity (CMI) associated with aging is thought to be responsible for these increased rates. Similarly, the decreased level of CMI among persons with malignancies and HIV infection is thought to be responsible for higher rates of shingles among them. Approximately 4% of individuals will experience a second episode of shingles [3].

Postherpetic neuralgia is the most challenging and debilitating complication of herpes zoster in the immunocompetent host [6]. Persistent pain after disappearance of the skin rash at the involved dermatome, known as postherpetic Neuralgia (PHN), can develop and is seen more frequently in older cases. Pain that persists or appears more than 90 days after the onset of rash is a commonly accepted definition for PHN [7]. On average, PHN lasts from three to six mo,nths but can persist longer. The severity of pain can vary and may be constant, intermittent, or triggered by stimulation of the affected area, such as wind on the face [8].

In this research we studied clinical characteristics of herpes zoster in patients who are admitted in a teaching referral hospital in north of Iran.

## 2. Method

This is a prospective-descriptive study about clinical symptoms, signs, and complications of 132 shingles patients who are admitted in a referral teaching hospital in north of Iran with one-year followup. Our sampling method was accessible sampling. Clinical criteria which we considered as shingles were any person with at least one of the following two: abnormal skin sensations with an acute onset of localized maculopapulovesicular unilateral rash, involving at least one dermatome (Zona), or an acute onset of disseminated maculopapulovesicular rash, beyond the involvement of one dermatome.

We started this study in January 2007 and recorded herpes zoster patient's data and followed them in two episodes, 3 months and 12 months after discharge from our hospital. This study continued until December 2013. A general physician and a nurse called the patients and recorded the data. Then, the collected data were processed using SPSS software (ver. 13).

## 3. Result

132 patients were admitted in Razi Hospital (therapeutic center of infectious diseases in north of Iran) in Mazandaran province, during 6 years from 2007 to 2013, with shingles impression.

52 cases (39.6%) were female and 80 (60.4%) were male. Mean age was 56.3 ± 20.1.

**Figure 1:**
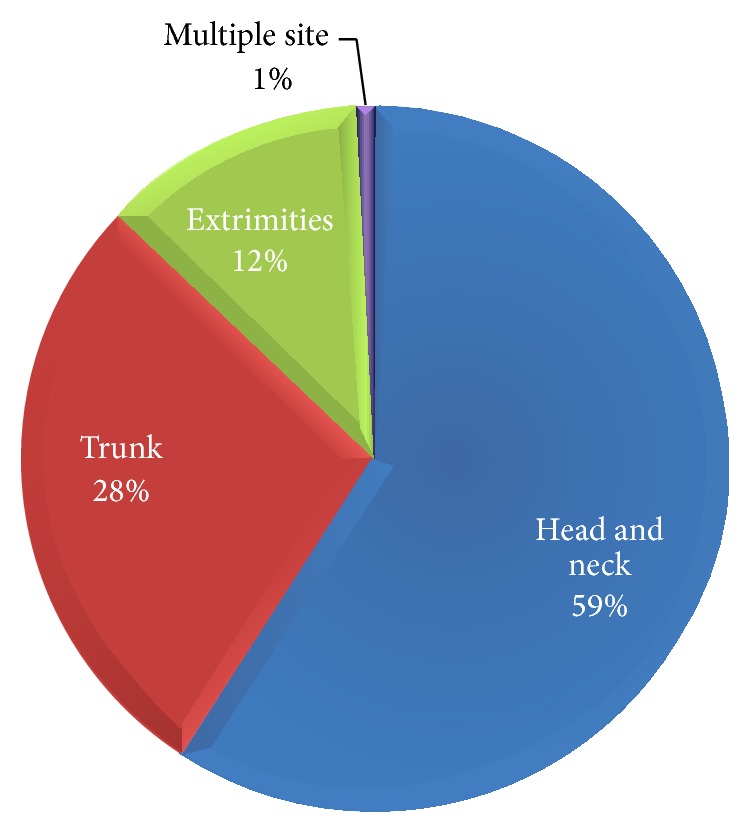
Percent of anatomical distribution of rashes.

126 cases (95.5%) had past history of chickenpox. The average time from the beginning of symptoms to admission was 5.7 days (SD = 4.240). Head and neck involvement occurred in 78 cases (59.1%), trunk involvement in 37 cases (28%), and extremity involvement in 16 cases (12.1%), and one patient (0.8%) had systemic presentation (Figure 1).

**Figure 2:**
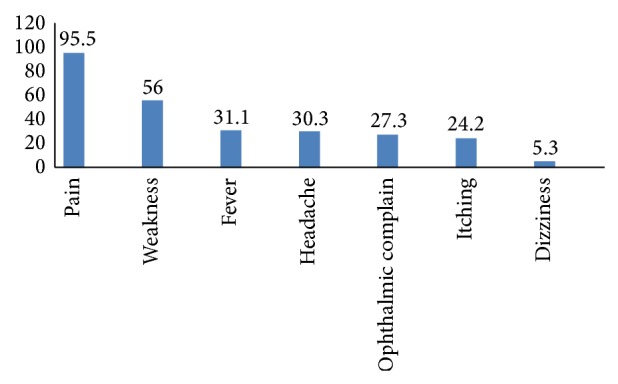
Prevalence of the most common symptoms (percent).

The most common symptoms were pain (95.5%), weakness (56%), fever (31.1%), headache (30.3%), ophthalmic complaints (27.3%), itching (24.2%), and dizziness (5.3%) (Figure 2).

**Figure 3:**
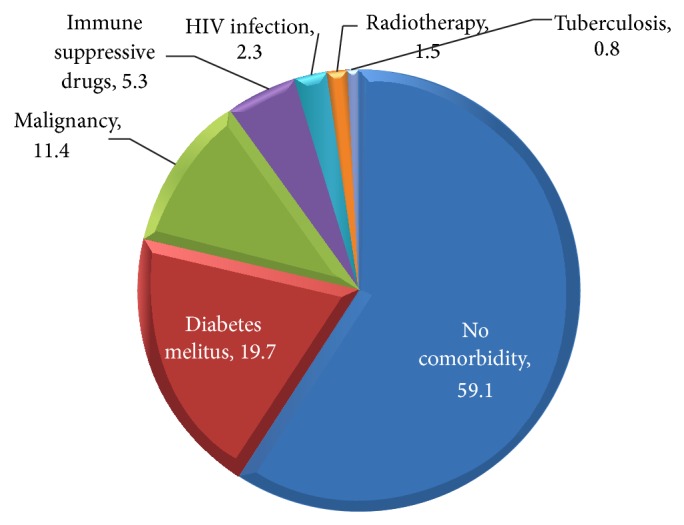
The prevalence of comorbidities in patients admitted with herpes zoster (percent).

Although 78 patients (59.1%) had no any comorbid disease, 54 cases (40.9%) had predisposing factors including diabetes mellitus in 26 cases (19.7%), malignancy in 15 cases (11.4%), immunosuppressive medication in 7 cases (5.03%), HIV infection in 3 cases (2.3%), radiotherapy in 2 cases (1.5%), and tuberculosis in one (0.8%) (Figure 3).

21 cases (15.9%) had bacterial superinfection blistering areas and overall 18 cases (13.6%) had opium addiction. 4 cases (3.03%) died during admission because of comorbidities. Postherpetic neuralgia was reported in 56 people (42.5%) in three months of followup and seven cases (5%) in one-year followup.

## 4. Discussion

Varicella infection is a prerequisite for the development of shingles. In temperate climates in the absence of a varicella vaccination program, the lifetime risk for varicella infection is over 95% [9]. In our study more than 95% of patients had positive history of chickenpox.

PHN is observed in 9–45% of all cases of herpes zoster (HZ) and the incidence has been reported to be as high as 50–60% among elderly or immunosuppressed patients [10, 11]. In this study postherpetic neuralgia was reported in 56 cases (42.5%) in three-month followup and seven cases (5%) in one-year followup.

The dermatomes from T3 to L3 are most commonly involved in HZ [12] but in this study head and neck involvement was most common and occurred in 78 cases (59.1%) and other involved sites were trunk in 37 cases (28%) and extremities in 16 cases (12.1%), respectively, and one person (0.8%) had systemic manifestation with head and neck, trunk, and extremities dermatomal presentation.

The incidence rate of herpes zoster ranges from 1.2 to 3.4 per 1,000 person/year among younger healthy individuals, increasing to 3.9–11.8 per 1,000 person/year among those older than 65 years, and incidence rates worldwide are similar [13, 14]. This relationship with age has been demonstrated in many geographical areas [13–18] and is attributed to the fact that cellular immunity declines as people grow older. In our study patients' age was between 36.2 and 76.4 years with average of 56.3 years.

Although some studies showed that the risk is increased in females [19], in this research most of the patients were male (80 cases (60.4%)) and 52 cases (39.6%) were female.

Another important risk factor is immunodeficiency, such as HIV infection [20, 21]. 2.3% of patients were HIV positive in our study with CD4 count greater than 200 and none of these patients developed PHN.

In our research all the patients were Caucasian and we cannot compare to other cases. However according to a study in North Carolina, black people were significantly less likely to develop zoster than white subjects [22].

Other potential risk factors include exposure to immunosuppressive drugs and immunotoxins [23]. We also find that radiotherapy and immunosuppressive therapy are major risk factors for HZ especially in old age.

There is no strong evidence for a genetic link or a link to family history. A study performed in 2007 showed that people with close relatives who had had shingles were twice as likely to develop Zona [23], but another study in 2010 found no such link [24] and in our study we did not find any link between relatives.

22.1% of patients in our study had herpes zoster ophthalmicus. This is like other studies. Herpes zoster ophthalmicus involves the ophthalmic branch of the trigeminal nerve and occurs in approximately 10% to 25% of cases [25].

Regarding previous studies in the world varicella vaccination has excellent effect for decreasing Zona and PHN [26–28]. Unfortunately we do not have this strategy in our region and the best way for decreasing Zona and PHN and other morbidities is immediate referring to the physician and quick starting of antiviral agent and probably steroids.

In this study average time before treatment from the beginning of the symptoms was 5.7 days (std. deviation = 4.240) and this time is too late for improvement after medical therapy and probably for decreasing of complication. If they refer to specialists in first 72 hours and receive antiviral and corticosteroid therapy it helps to decrease duration of the disease, accelerate improvement of the lesions, and decrease symptoms such as pain and may decrease PHN [18] and other complications such as superinfection.

## 5. Conclusion

It seems that more delay for visiting by physician can result in more complications, especially PHN. For improving this we need more education to community and general practitioners.
